# The diagnostic accuracy of the MyDiagnostick to detect atrial fibrillation in primary care

**DOI:** 10.1186/1471-2296-15-113

**Published:** 2014-06-09

**Authors:** Bert Vaes, Silke Stalpaert, Karen Tavernier, Britt Thaels, Daphne Lapeire, Wilfried Mullens, Jan Degryse

**Affiliations:** 1 Department of General Practice, Katholieke Universiteit Leuven (KUL), Leuven, Belgium; 2 Institute of Health and Society, Université Catholique de Louvain (UCL), Brussels, Belgium; 3 Department of Cardiology, Ziekenhuis Oost Limburg, Genk, Belgium; 4 Biomedical Research Institute, Faculty of Medicine and Life Sciences, Hasselt University, Diepenbeek, Belgium

**Keywords:** Atrial fibrillation, Sensitivity and specificity, MyDiagnostick, Primary care

## Abstract

**Background:**

Atrial fibrillation is very common in people aged 65 or older. This condition increases the risk of death, congestive heart failure and thromboembolic conditions. Many patients with atrial fibrillation are asymptomatic and a cerebrovascular accident (CVA) is often the first clinical presentation. Guidelines concerning the prevention of CVA recommend monitoring the heart rate in patients aged 65 or older. Recently, the MyDiagnostick (Applied Biomedical Systems BV, Maastricht, The Netherlands) was introduced as a new screening tool which might serve as an alternative for the less accurate pulse palpation. This study was designed to explore the diagnostic accuracy of the MyDiagnostick for the detection of atrial fibrillation.

**Methods:**

A phase II diagnostic accuracy study in a convenience sample of 191 subjects recruited in primary care. The majority of participants were patients with a known history of atrial fibrillation (n = 161). Readings of the MyDiagnostick were compared with electrocardiographic recordings. Sensitivity and specificity and their 95% confidence interval were calculated using 2x2 tables.

**Results:**

A prevalence of 54% for an atrial fibrillation rhythm was found in the study population at the moment of the study. A combination of three measurements with the MyDiagnostick for each patient showed a sensitivity of 94% (95% CI 87 – 98) and a specificity of 93% (95% CI 85 – 97).

**Conclusion:**

The MyDiagnostick is an easy-to-use device that showed a good diagnostic accuracy with a high sensitivity and specificity for atrial fibrillation in a convenience sample in primary care. Future research is needed to determine the place of the MyDiagnostick in possible screening or case-finding strategies for atrial fibrillation.

## Background

Atrial fibrillation is currently the second most frequently occurring cardiac arrhythmia in clinical practice (after extrasystole). Its prevalence is estimated around 0.4 – 1.0% in the general population, increasing with age, up to 5.0 – 6.0 in those aged 65 or older and 8.0% in subjects aged 80 or older [1].

More than 30% of patients with atrial fibrillation are asymptomatic. Furthermore, this condition increases the risk of death, congestive heart failure and thromboembolic conditions [2,3]. The risk of cerebrovascular accident (CVA) even increases fivefold in case of atrial fibrillation and CVA is often the first clinical presentation of atrial fibrillation. Moreover, the risk for CVA increases with increasing age; from 9.9% in patients between 70 – 79 years to 24% in the group of patients between 80 – 89 years [4-6].

The current European guidelines on atrial fibrillation emphasize the importance to start anticoagulation in order to decrease the risk of CVA [6]. In order to assess this risk, the CHADS_2_ score is used, along with the more recent and more accurate CHA_2_DS_2_VASc score [7,8]. Therefore, the high prevalence of atrial fibrillation, the potentially serious consequences of the condition, the presence of effective therapy and the minimal impact of treatment on the quality of life of the patient ensure that atrial fibrillation is a condition for which screening can or should be done [9].

**Figure 1:**
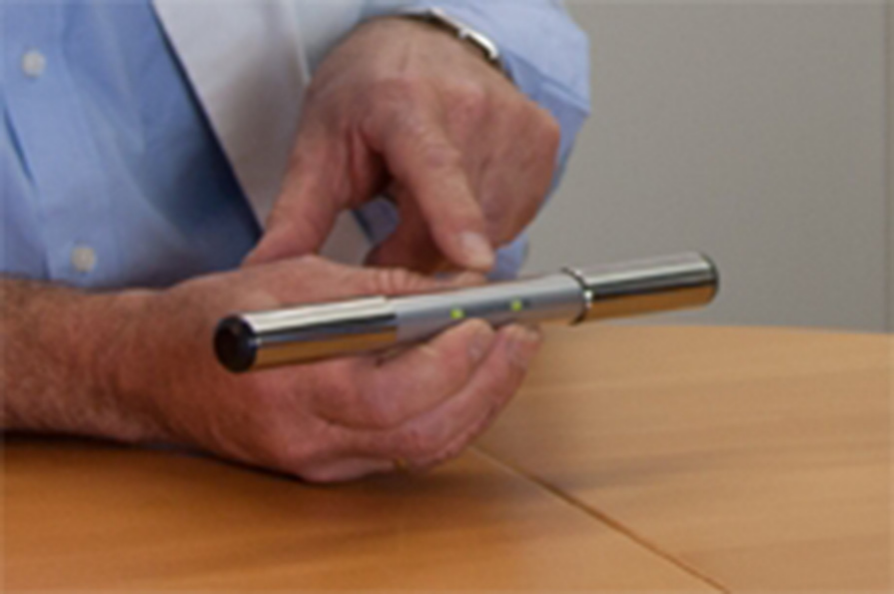
The MyDiagnostick device.

Guidelines concerning the prevention of CVA recommend monitoring the heart rate in patients aged 65 or older, followed by electrocardiogram (ECG) monitoring in case of an irregular rhythm [6,10]. Palpation of the pulse, to monitor the heart rate, has a good sensitivity but a low specificity, making this a useful method of ruling out atrial fibrillation but a less good method of confirming it [11]. Recently, the MyDiagnostick (Applied Biomedical Systems BV, Maastricht, The Netherlands) [12] was introduced as a new screening tool and an alternative for the less accurate pulse palpation. This device has the form of a rod with a metal handle on both ends. There are electrodes in these handles making it possible to record a single-lead ECG (Figure 1). This new screening technique would facilitate home screening and increase the likelihood of detection of asymptomatic atrial fibrillation.

Therefore, this study was designed to explore the diagnostic accuracy of the MyDiagnostick for the detection of atrial fibrillation.

## Methods

### Study population

Participating general practitioners were asked to invite patients with known, paroxysmal or chronic atrial fibrillation to participate in the study. Furthermore, this convenience sample was added up with subjects without a history of atrial fibrillation. With the probability of finding a false positive result of 5% or less (α = 0.05), an estimated prevalence of atrial fibrillation of 50% in the study population, an expected sensitivity and specificity of 95% and a confidence interval of 4%, a sample size of 160 subjects was needed [13]. To end up with a prevalence of atrial fibrillation of at least 50% at the moment of the study the vast majority of the invited patients were people with a known history of atrial fibrillation (161/191) and only 30 people without a history of atrial fibrillation. The patients who were willing to take part in the survey were given an appointment for an examination by the research team in their own general practice centre. Before participating in the study, all patients signed an informed consent. The study was approved by the ethical review board of the Medical Faculty of the Catholic University of Leuven, Belgium (no ML 10464). Patients wearing a pacemaker were excluded if the pacemaker was configured in active pacing mode.

Clinical characteristics were registered by the researcher (SS, KT, BT or DL). The body mass index was calculated, the blood pressure measured and a limited number of comorbidities extracted from the electronic medical record. The presence of diabetes mellitus type II, arterial hypertension and peripheral arterial illness and a history of coronary heart disease and transient ischaemic attack or CVA was reported and the CHA_2_DS_2_VASc score for each participant calculated. The intake of platelet aggregation inhibitors or anticoagulants (warfarin or new oral anticoagulants) was registered.

### A trial fibrillation

**Figure 2:**
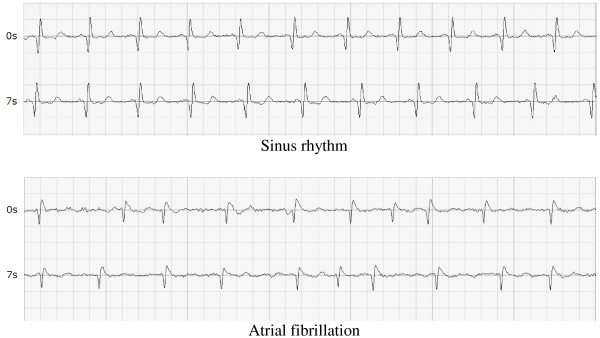
Single-lead ECGs provided by the MyDiagnostick (sinus rhythm and atrial fibrillation).

Each participant was tested with the MyDiagnostick (Applied Biomedical Systems BV, Maastricht, The Netherlands) [12] by a single researcher who was not blinded for the medical history of the patient. This device has the form of a rod with a metal handle on both ends. In these handles electrodes make it possible to record a single-lead ECG that is analyzed automatically. The patient was asked to grasp the device by both handles. After one minute the ECG lead was analysed and LED indicators gave a red or green signal that could be interpreted as the presence or absence of atrial fibrillation. Three consecutive recordings with the MyDiagnostick with a 1 – 2 minute interval were done. Figure 2 shows single-lead ECGs provided by the MyDiagnostick. The MyDiagnostick has a storage capacity of 140 recordings of 50 – 70 seconds.

The method of detection of AF in the MyDiagnostick is based on the measurement of R-R interval irregularity. Prior AF detection, the acquired ECG (1 minute) is pre-processed and R-waves are detected. From all detected R-wave annotations, R-R intervals are computed and used as an input for AF detection. The AF algorithm calculates an overall AF score based on a base rhythm-, periodicity- and variability score. The base rhythm score is based on a normal sinus rhythm state-machine chaining normal R-R intervals, including occasional premature intervals and short runs of tachycardia. Creation of long chains reflects a fit of the sinus rhythm state-model, lowering the probability of AF. The periodicity and variability scores are based on the R-R autocorrelation function. Periodicity of R-R interval patterns will generate multiple correlation peaks, whereas R-R interval irregularity will lower correlation at only a small shift. The overall AF score is obtained by linear combination of all scores and compared to a threshold, producing a dichotomous result (AF/no AF).

Afterwards a 12-lead electrocardiogram (ECG) (gold standard) was carried out once by the same researcher. The ECGs were done using digital machines (HeartScreen 80 G-L (MediSafe Ltd, Winchester, United Kingdom), Welch Allyn CP 50 (Welch Allyn, New York, USA), Nihon Kohden Cardiofax S 1250 (Nihon Kohden, Tokyo, Japan) or Schiller AT101 (Schiller AG, Baar, Switzerland)) and the data were immediately printed. The ECGs were analyzed off-line on the basis of the Minnesota Code Classification System for Electrocardiographic Findings [14] (code 8-3-1) by an experienced cardiologist (WM), blinded for the software interpretation and the results from the MyDiagnostick.

The readings of the MyDiagnostick were compared with the electrocardiographic recording. The overall three measurements on the MyDiagnostick were viewed for each patient. A green light three times was interpreted as a negative result and a red light three times as a positive result. The non-uniform results of the MyDiagnostick were interpreted in favour of the most common outcome (i.e. 2x red and 1x green was interpreted as a positive result, while 1x red and 2x green was interpreted as a negative result).

### Statistical analysis

Sensitivity and specificity and their 95% confidence interval were calculated using 2x2 tables (MedCalc^®^ 11.6.0.0 (Mariakerke, Belgium)). The positive and negative predictive values were then estimated based on an expected prevalence of atrial fibrillation of 6% in the population aged 65 or older.

## Results

**Figure 3:**
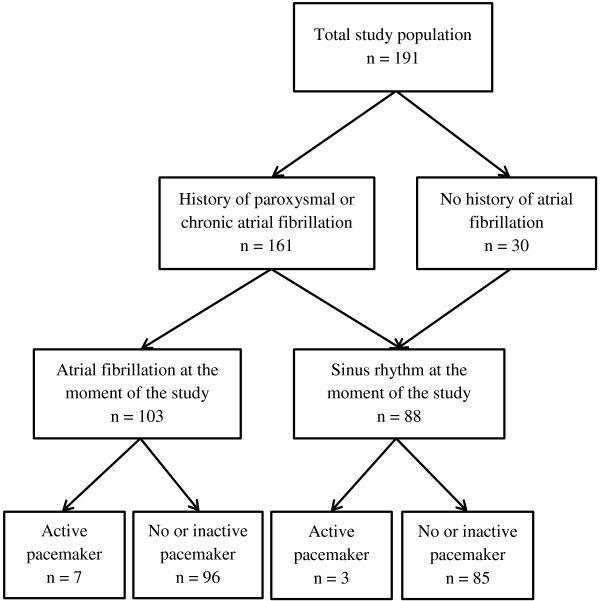
Flow chart of study participants.

A total of 191 subjects participated in this study of which 91 (48%) women. The mean age of the participants was 74.6 ± 9.7 years (range 50 – 99 years). In total 30 participants without a history of atrial fibrillation (all in sinus rhythm on ECG) and 161 participants that were known to have paroxysmal or chronic atrial fibrillation were included (Figure 3). Of these latter subjects, 103 showed atrial fibrillation on their ECG at the moment of the study. Thus, a prevalence of 54% for an atrial fibrillation rhythm was found in the study population. Table 1 shows the patient characteristics according to the presence of atrial fibrillation. A pacemaker was present in 17 participants, in 10 patients the pacemaker was active at the moment of the ECG recording and they were therefore excluded from further analysis. The analysis was continued with 181 participants (prevalence of atrial fibrillation, 53%).

**Table 1:** Characteristics of the study population (n = 191)

	**Atrial fibrillation present**	**Atrial fibrillation absent**
	**(n = 103)**	**(n = 88)**
Age, mean ± SD	77 ± 8	71 ± 11
Male gender, n (%)	57 (55.3)	43 (51.1)
BMI, mean ± SD	27.7 ± 6.9	25.9 ± 6.5
Systolic blood pressure, mean ± SD	130 ± 19	130 ± 18
Diastolic blood pressure, mean ± SD	75 ± 14	75 ± 10
CHA_2_DS_2_VAS_C_ score, median (IQR)	3 (2 – 4)	3 (2 – 3)
Comorbidity		
Diabetes mellitus type II, n (%)	26 (25.2)	15 (17.0)
Arterial hypertension, n (%)	94 (91.3)	62 (70.5)
Coronary heart disease, n (%)	9 (8.7)	16 (18.2)
TIA or CVA, n (%)	17 (16.5)	4 (4 .5)
Peripheral arterial illness, n (%)	4 (3.9)	4 (4.5)
Anticoagulation		
No anticoagulation, n (%)	0 (0)	22 (25.0)
Platelet aggregation inhibitor, n (%)	6 (5.8)	24 (27.3)
Warfarin, n (%)	69 (67.0)	30 (34.1)
New oral anticoagulants, n (%)	28 (27.2)	12 (13.6)

SD: standard deviation; IQR: inter-quartile range; TIA: transient ischaemic attack; CVA: cerebro-vascular accident.

The MyDiagnostick showed a positive result in 96 participants and a negative result in 85 subjects (Table 2). No unsuccessful attempts were reported. In the event of a uniform result (n = 166, 92%) the diagnosis was correct in 96% of cases (seven misdiagnoses, four false positives and three false negatives). A total of 15 participants showed non-uniform results (nine positive results, and six negative results), in which 10 participants were interpreted correctly (seven true positives and three true negatives) and five were misinterpreted. On the basis of these results sensitivity for the MyDiagnostick of 94% (95% CI 87–98) and a specificity of 93% (95% CI 85–97) was obtained. Based on an expected prevalence of 6% in the population aged 65 or older a positive predictive value of 45% (95% CI 24 – 68) and a negative predictive value of 99% (95% CI 97 – 100) were estimated.

**Table 2:** 2x2 table to calculate the diagnostic accuracy of the MyDiagnostick

	**Atrial fibrillation present**	**Atrial fibrillation absent**	**Total**
MyDiagnostick positive result	90	6	96
MyDiagnostick negative result	6	79	85
Total	96	85	181

## Discussion

In this study of a convenience sample in primary care, the MyDiagnostick was able to accurately set the diagnosis of atrial fibrillation with a high sensitivity and specificity. The sensitivity measures the proportion of actual positives that have been correctly identified and the specificity measures the proportion of the actual negatives that have been appropriately recognized as such. These two measures are closely related to the concepts of type I (a test that shows a patient to have a disease when in fact the patient does not have the disease) and type II (a test that shows a patient not to have a disease when the patient does have the disease) errors. A perfect diagnostic test would be 100% sensitive (no false negatives) and 100% specific (no false positives). However, in a low prevalence setting like primary care and for possible screening purposes the sensitivity of a test, the capacity to rule out the disease, could be considered as the most important measure.

The results for the MyDiagnostick are in line with the diagnostic accuracy found for other screening methods and devices. Pulse palpation to detect an irregular heartbeat gave a high sensitivity (87% – 97%) but a moderate to low specificity (71% – 81%) [11,15]. Although the specificity could possibly be improved by continued pulse registration, but at the cost of decreasing sensitivity. The Microlife BP monitor (Microlife Corporation, Taipei, Taiwan), a modified sphygmomanometer that records pulse intervals, gave a sensitivity of 100% and a specificity of 90% (95% CI 87 – 92) when multiple measurements were carried out [16]. Another study that also used a modified sphygmomanometer showed a sensitivity of 95% (95% CI 93 – 98) and specificity of 86% (95% CI 84 – 98) in a single measurement and a sensitivity of 97% (95% CI 91 – 99) and specificity of 89% (95% CI 85 – 92) on 3 measurements [17]. A finger probe that registered a pulse wave pattern for 30 seconds gave a sensitivity of 100% and a specificity of 91% [18]. Two studies, similar to the current study, also used a device that registered a single-lead ECG in which the interpretation was done afterwards by a primary care physician [17,19]. Somerville et al. found a sensitivity of 96% (95% CI 80 – 100) and a specificity of 98% (95% CI 91 – 100) [17], and Mant et al. found a sensitivity of 85% (95% CI 79 – 91) and a specificity of 86% (95% CI 85 – 88) with the use of a thoracic lead and a sensitivity of 83% (95% CI 75 – 89) and a specificity of 89% (95% CI 87 – 90) with the use of a peripheral lead [19]. The latter sensitivities and specificities were moderate primarily due to the varying interpretation of ECGs by primary care physicians. By contrast, the advantage of the MyDiagnostick is the absence of an interpretation bias.

This study was a phase II diagnostic study investigating the accuracy of the MyDiagnostick in a convenience sample of healthy people and people with a history of atrial fibrillation. However, before a clinical recommendation on the use of the MyDiagnostick in every day clinical practice can be given, a phase III diagnostic study has to be performed, determining the clinical consequences of introducing the MyDiagnostick through a randomised trial. The place of the MyDiagnostick in possible future screening programs for atrial fibrillation therefore remains to be determined. The use of the MyDiagnostick seems much more practical than electrocardiographic monitoring at home. It involves no skin electrodes or wires and the use of the device does not require any experience or medical knowledge. Furthermore, using the MyDiagnostick is faster and cheaper. The MyDiagnostick also has an additional advantage in comparison with a blood pressure monitor that detects an irregular pulse, since every recording is registered and the single-lead ECGs can be consulted later on to confirm the red lights that the patient detected.

In terms of user friendliness, this screening tool is easily manageable in the population aged 65 or older. The patient only needs to hold the ends of the rod with both hands. The unit goes on automatically, so that a visual limitation cannot be a restricting factor. Furthermore, holding with the palm of the hand up or down, holding it lightly or firmly, trembling and physical activity during use do not seem to influence the accuracy and uniformity of the results.

In addition to detecting paroxysmal atrial fibrillation and initiating the necessary treatment, the following advantages of future use could be hypothesized: following up on the response to electroconversion and medical treatment, improving compliance with therapy and potentially decreasing medical costs (by means of the prevention of ischemic CVA).

### Strengths and limitations

This is the first study investigating the diagnostic accuracy of the MyDiagnostick in primary care. High sensitivities and specificities were obtained in the study population, making this easy-to-use device a possible candidate to implement in future screening or case-finding programs for atrial fibrillation. However, a few limitations of the current study should be mentioned. First, a prevalence of atrial fibrillation of 6% was assumed in subjects aged 65 and older. However, a variety of prevalence figures is found in literature and is mainly the result of differences in the study population, the methods used (Holter, serial ECGs …) and the duration of ECG recording. Second, several ECG devices were used as the gold standard instead of one standardized device. Third, the possible place of the MyDiagnostick in a screening or case-finding strategy was not determined with the current study, nor was a cost-benefit analysis of a screening or case-finding strategy with the MyDiagnostick done. And fourth, because the study population was a convenience sample extrapolation of these results to the general population should be made with caution. Furthermore, investigations concerning the reliability of the MyDiagnostick for other arrhythmias and test-retest reliability must still be done.

## Conclusion

The MyDiagnostick is an easy-to-use device that showed a good diagnostic accuracy with a high sensitivity and specificity for atrial fibrillation in a convenience sample in primary care. Future research is needed to determine the place of the MyDiagnostick in possible screening or case-finding strategies for atrial fibrillation.

## Abbreviations

CVA: Cerebrovascular accident; CI: Confidence interval; ECG: Electrocardiogram; SD: Standard deviation; IQR: Inter-quartile range; TIA: Transient ischaemic attack.

## Competing interests

The author declares that they have no competing interests.

## Authors’ contributions

BV, SS, KT, BT, DL drafted the manuscript. JD is responsible for the design, conduct and analysis of the study. All authors participated in the critical revision of the manuscript. All authors read and approved the final manuscript.

## Pre-publication history

The pre-publication history for this paper can be accessed here:

http://www.biomedcentral.com/1471-2296/15/113/prepub
